# Combination of Phytoactives in the Diet of Lactating Jersey Cows: Effects on Productive Efficiency, Milk Composition and Quality, Ruminal Environment, and Animal Health

**DOI:** 10.3390/ani14172518

**Published:** 2024-08-29

**Authors:** Maksuel G. de Vitt, Mateus H. Signor, Natalia G. Corrêa, Michel Breancini, Gabriel J. Wolschick, Bruna Klein, Luiz Eduardo L. Silva, Roger Wagner, Camila T. K. Jung, Gilberto V. Kozloski, Miklos M. Bajay, Gabriela S. Schroeder, Carine F. Milarch, Aleksandro S. Da Silva

**Affiliations:** 1 Graduate Program in Animal Science, Universidade do Estado de Santa Catarina (UDESC), Chapecó 89815-630, SC, Brazil; mak-witt@hotmail.com (M.G.d.V.); miklos.bajay@udesc.br (M.M.B.); 2 Department of Animal Science, Universidade do Estado de Santa Catarina (UDESC), Chapecó 89815-630, SC, Brazil; mateus.signor@edu.udesc.br (M.H.S.); natalia.gc703@gmail.com (N.G.C.); michelbreancini11@gmail.com (M.B.); gabriel.jw@edu.udesc.br (G.J.W.); brunaklein06@yahoo.com.br (B.K.); 3 Department of Food Science, Universidade Federal de Santa Maria (UFSM), Santa Maria 97105-900, RS, Brazil; eduardo.lobo@acad.ufsm.br (L.E.L.S.); rogerwag@gmail.com (R.W.); 4 Department of Animal Science, Universidade Federal de Santa Maria (UFSM), Santa Maria 97105-900, RS, Brazil; camila.jung@acad.ufsm.br (C.T.K.J.); gilberto.kozloski@ufsm.br (G.V.K.); 5 Faculty IELUSC, Joinville 89201-270, SC, Brazil; gabisansch@gmail.com (G.S.S.); carine.milarch@ielusc.br (C.F.M.)

**Keywords:** dairy cattle, phytobiotic, nutraceutical, immunity, *Streptococcus*, animal health

## Abstract

**Simple Summary:**

Adding the phytoactive mixture to the dairy cows’ diet positively affected animal nutrition and health, improving Jersey production efficiency. The intake of phytogenics improved milk quality, reduced somatic cell counts, and increased unsaturated fatty acids. The intake of phytogenics per cow protected the mammary gland from bacteria *Streptococcus* spp. The phytoactive mixture directly affected the digestibility and modulation of the volatile fatty acid profile. The phytoactive mixture positively affected immune and antioxidant responses and anti-inflammatory action.

**Abstract:**

This study’s objective was to evaluate whether adding a combination of phytoactive (microencapsulated essential oils, minerals, turmeric extract, tannin, prebiotic, and probiotic) to the feed of lactating Jersey cows positively affects the production, composition, and quality of milk, rumen environment, and animal health. Fourteen Jersey cows were divided into two groups (control and phytogenic) for an experiment with two lactation phases of 45 days each (early lactation and mid-lactation). During the experiment, milk production was higher at various times in cows that consumed phytoactive, and these animals had the best feed efficiency. In mid-lactation, phytoactive intake increased nutrient digestibility. The number of lymphocytes in the blood is reduced when cows consume phytoactive substances. Globulin levels increased in these cows fed with the additive, which may be related to a higher concentration of immunoglobulins, especially IgA. Cows fed phytoactives had lower ceruloplasmin and haptoglobin concentrations. Lower serum lipid peroxidation, associated with greater glutathione S-transferase activity, is a good health indicator in cows that consume phytoactive substances. The higher concentration of volatile fatty acids was due to the higher proportion of acetic acid in the ruminal fluid combined with lower butyric acid. Somatic cell counts in milk were lower in cows that consumed phytoactives during mid-lactation, as well as the effect of the treatment on *Streptococcus* spp. (lower in cows that consumed the additive). We conclude that consuming the additive benefits cows’ health modulates rumen fermentation and nutrient digestibility, and positively affects milk production and quality.

## 1. Introduction

Milk production has grown considerably over the last ten years [1]; however, the number of cows milked has not seen a representative increase in productivity, which shows that dairy farms are becoming more efficient. Dairy cow nutrition influences milk production and quality, cows’ health, and the system’s environmental impacts. The symbiosis between ruminants and microorganisms is fundamental; the microorganisms degrade the feed consumed and provide the animals mainly with fatty acids and microbial protein; however, in this process, heat, ammonia, and methane are produced, which are considered protein and energy losses [2]. To reduce these losses and consequently enhance animal performance by improving efficiency in using feed, alternatives have been explored, such as additives in the diet [3].

Cows at peak production have high metabolic demands and are more prone to disorders such as physiological oxidative stress and decreased immune response [4]. Natural feed additives are on the rise due to their numerous benefits to animals, including antioxidant, anti-inflammatory, antibacterial, antiviral, coccidiostats, and anthelmintic actions [5,6,7]. In addition to having great immunological functionality, some natural compounds act in rumen fermentation to improve nutrient absorption and increase ruminant productivity [8,9]. These actions can improve cows’ health by avoiding or minimizing metabolic disorders and infectious diseases such as mastitis, thus maintaining constant productivity. In cases of mastitis, due to the antimicrobial action, there has been a record of positive effects of cinnamon and oregano essential oils [10,11] and curcumin [12], which are ingredients of the phytogenic used in the present study.

It is essential to note that the work carried out has shown the efficiency of phytogenics in a mixed and individual way. Despite this, research is needed to adjust indications, dosages, and supply modes so adverse reactions do not occur, according to the literature [13]. Commercial products, defined as blends, have gained space in the animal nutrition market, where combinations of essential oils, plant extracts, organic minerals, prebiotics, and probiotics in search of synergism and health and productive efficiency are becoming an increasingly common practice. However, in these combinations, little is known scientifically about the real benefits of including these additives in the diet for lactating cows; at the same time, there is a need to avoid or minimize health problems in high-producing cows, which are constantly challenged.

We hypothesized that cows that consume the additive produce more milk and milk with lower somatic cell count (SCC) because the synergism between the ingredients present in the additive would trigger an antioxidant and modulate ruminal fermentation. Therefore, this study’s objective was to evaluate whether adding a combination of phytoactives to the feed of lactating primiparous Jersey cows positively affects milk production, composition and quality, rumen environment, and animal health.

## 2. Materials and Methods

### 2.1. Additive

The product used was a commercial additive (Phytomast^®^ Concentrado, Tecphy, Canelinha, Brazil). Its formulation is based on cinnamon and oregano essential oil, chelated amino acid chromium (min. 200 mg/kg), selenium proteinate (min. 250.00 mg/kg), inactivated *Saccharomyces cerevisiae* (min. 270 g/kg), *S. cerevisiae* (min. 5.0 × 10^11^ CFU/kg), turmeric extract, and tannic acid. The dosage used was 20 g per animal day, as recommended by the manufacturer.

### 2.2. Animals and Facilities

The experiment was conducted in the ruminant sector—dairy cattle farming at the Experimental Farm (FECEO) of the State University of Santa Catarina (UDESC) located in Guatambu/SC, Brazil. Fourteen primiparous Jersey cows, 27 ± 0.5 months old and weighing 410 ± 12.6 kg, with 30 days in milk (DIM), were used. The cows used in the research were daughters of the same bull (Swoosh, STgenetics, Indaiatuba, SP, Brazil) to have a consistent genetic standard for the experiment. Animals were acquired by the university shortly after birth, where they were managed and fed similarly until the current experimentation phase. At the experiment’s beginning, the cows’ body score was 3.0 to 3.5, indicating a homogeneous herd.

The herd was housed in a compost barn-type sawdust bed confinement system, with 20 m^2^ per animal. The animals were milked using two types of milking systems: (a) a mechanical system in a herringbone milking parlor (45 days) and (b) a robotic milking system in a guided flow system (45 days).

In the pre-partum period, 60 days before the start of the experiment, the animals received a vaccine for clostridiosis and underwent parasite control (ticks, flies, and helminths). Pre-partum, the animals received an anionic diet. Still, in the first stage of the experiment, one of the cows in the control group became ill (bovine parasitic disease), and despite treatment, the cow died. Thus, 13 cows (6 and 7) remained for the control and treated groups, respectively.

### 2.3. Experimental Design and Diets

#### 2.3.1. Early Lactation—Peak Lactation

The early lactation phase lasted 45 days (15 days of adaptation + 30 days of data collection), during which the animals were randomly divided into two groups of seven cows: phytogenic, where the concentrate had 20 g of Phytomast^®^ (feed additive) added, and control, which received the basal concentrate without added additives. To form the groups, we considered the body score, body weight (control: 412 ± 12.8 kg; phytogenic: 408 ± 11.9 kg), and the average milk production of these cows over the last seven days before the beginning of this experiment (control group: 19.8 ± 0.56 kg of milk; phytogenic group: 20.0 ± 0.52 kg of milk).

The diets were formulated according to the nutritional requirements of the animals [14] with a 52 V:48 C ratio, considering the following feeds: concentrate, Tifton 85 hay, and corn silage, which were mixed and supplied in the form of a total mixed ration (TMR) in individual feeders (with the animals restrained), divided into three daily treatments (06:00, 11:00, and 16:00). Water was provided ad libitum in the shed and during feeding when cows were contained by a cage (that is, a drinking trough separated the feeders) (Supplementary Materials S1). It is essential to make it clear that each cow was fed in its feeder, i.e., the feeding was individual for each animal despite being in a compost barn system. The composition of the concentrate and the feeds used in the diet are shown in Table 1.

In early lactation, the cows were milked mechanically at 7:00 a.m. and 4:30 p.m., with three milking sets using automatic extraction [model AMI 5550 (Boumatic^®^, Moncton, NB, Canada)].

#### 2.3.2. Mid-Lactation

After completing the first stage, the animals continued to consume the same diet as in early lactation for 15 days (with and without the phytogenic, according to their group). This interval was not foreseen in the initial project but was necessary because the installation of the robotic milking system had yet to be completed.

Next, mid-lactation began, which also lasted 45 days (15 days of adaptation + 30 days of data collection), where the division of animals was the same as in early lactation, with seven cows in each group: phytogenic, where the concentrate included 20 g of Phytomast^®^ (feed additive); control, which received the basal concentrate without added additives. It is essential to highlight that the same animals were always kept in each group; therefore, the cows in the phytogenic group consumed the feed additive for 105 continuous days (early lactation + 15-day interval + mid-lactation).

At this stage, the cows had a DIM of approximately 90 days; they would have the robot’s feed, and a new diet was formulated according to the nutritional requirements of the animals [14] with a 52 V:48 C ratio, considering the following feeds: concentrate, Tifton 85 hay and silage of corn. These feeds were mixed and fed into individual feeders, which were also divided into three daily treatments. Water was supplied ad libitum, as in early lactation. The composition of the concentrate and feeds used in the diet are also shown in Table 1.

In mid-lactation, the cows were milked using a robotic milking system with guided flow at two times: 5:30 a.m. and 3:30 p.m. Therefore, when milking was not scheduled, the robot remained in the “manual” function, with doors closed. At these milking start times, the “automatic” function was switched. After milking, the animals entered the feeding lane, waiting for feed to be supplied.

### 2.4. Productive Performance

Daily milk production was measured using a digital production marker coupled to the mechanical milking system during early lactation, and the output was recorded on a spreadsheet. In mid-lactation, automatic milking determines the daily production of each cow, with data stored on a computer connected to the robot. In two lactation phases, the amount of feed provided was measured, and after feeding, the amount of feed left over was weighed to determine daily consumption. Based on these data, dairy efficiency is calculated: milk production/feed consumption.

Feed samples were pre-dried in a forced ventilation oven at 55 °C for 72 h, then removed from the oven and weighed again to determine the partial dry matter content, followed by grinding in a Wiley-type mill (Marconi, model: MA340, Piracicaba, SP, Brazil) using a 1 mm mesh sieve. The pre-dried and ground samples were heated at 105 °C to obtain the MS and the mineral material in a muffle furnace at 600 °C [15]. The micro-Kjeldahl method determined the N content (Method 984.13, AOAC, 1997), which made it possible to predict the CP content through mathematical calculation. To determine the neutral detergent fiber (NDF) content, samples were placed in polyester bags [16] and treated with a neutral detergent solution in an autoclave at 110 °C for 40 min [17]. Acid detergent fiber (ADF) concentrations were determined according to AOAC (1997, method 973.18). The ether extract portion of the samples was quantified using an automatic SER158 Fat Extractor (VELP^®^ Scientifica, Usmate Velate, MB, Italy).

### 2.5. Sampling and Sample Collection

Blood samples were collected at intervals of 15 days during early lactation and mid-lactation (DIM 30, 45, 60, and 75 and DIM 90, 105, 120, and 135, respectively). Samples were collected in tubes with clot activator (4 mL) to separate blood serum and in tubes with 10% EDTA anticoagulant (4 mL) for blood count analysis. The material was stored in a thermal box until processing, which took up to 3 h.

In both milking systems, milk collectors allow a homogeneous, individual sample of the entire milking. Milk for proximate composition analysis was collected during morning milking at DIM 30, 45, 60, and 75 in early lactation and DIM 90, 105, 120, and 135 in mid-lactation. TSCC and proximate composition in early lactation and mid-lactation were measured at strategic moments. Milk collection for fatty acid profile was carried out at DIM 60 and 75 in early lactation and DIM 120 and 135 in mid-lactation. To evaluate the microbiota of the mammary/milk gland at three moments (DIM 30, 75, and 135), the teats were cleaned using a pre-dipping solution and dried with paper towels. The first three jets were removed from each teat using gloves to reduce environmental contamination. This material was frozen at −80 °C until the end of the experiment. Afterward, it was thawed and homogenized, and a sample was collected using a specific commercial kit for transporting and preserving biological material to the laboratory.

Ruminal fluid was collected at the end of each experimental period, with DIM 75 in early lactation and DIM 135 in mid-lactation. For collection, a silicone esophageal probe was connected to a vacuum system, allowing the collection of material with pH measured in less than one minute after collection; then, it was placed in plastic pots and kept cool in an ice box.

Feces were collected to determine nutrient digestibility coefficients on the last three days of the experiment: DIM 73, 74, and 75 in early lactation and DIM 133, 134, and 135 in mid-lactation. Feces were collected directly from the rectal ampoule three times daily.

### 2.6. Hemogram

Hematologic variables were obtained using EDTA blood tubes and analyzed with the VET3000 automatic hematologic analyzer (EQUIP^®^), which determines the number of leukocytes, lymphocytes, granulocytes, monocytes, erythrocytes, and platelets, as well as determining the concentration of hemoglobin (g/dL) and hematocrit (%).

### 2.7. Serum Biochemistry and Proteinogram

The samples collected in tubes without anticoagulant were centrifuged in a QUIMIS^®^ tube centrifuge at 8000 revolutions per minute (rpm) for ten minutes. Subsequently, the serum was stored in microtubes at −20 °C until analysis. In the blood serum, serum levels of total protein, albumin, urea, C-reactive protein (CRP), cholesterol, and fructosamine were evaluated, and the activity of the enzymes aspartate aminotransferase (AST) and gamma-glutamyltranspeptidase (GGT) was determined. For this, ANALISA^®^ kits were used in EQUIPIVET equipment (Zybio EXC-200^®^, Shenzhen, China). The concentration of globulins was calculated as total protein—albumin.

Sodium dodecyl sulfate–polyacrylamide gel electrophoresis was performed using mini gels (10 × 10 cm), as described by researchers [18]. The gels were stained with Coomassie blue and photographed to identify and quantify protein fractions using Labimage 1D software (Loccus Biotechnology, Cotia, SP, Brazil). A standard containing fractions with molecular weight between 10 and 250 KD (Kaleidoscope—Bio-Rad, Maynard, MA, USA) was used as a reference.

### 2.8. Oxidative Status

Firstly, the concentration of proteins in serum samples was determined using the literature methodology [19], using bovine albumin as the standard. A calibration curve was previously constructed with albumin, and subsequently, the absorbance of the samples was measured at 595 nm using cuvettes in a spectrophotometer. Then, the quantification of total proteins in the serum samples was performed, and the data were used to calculate the results of the oxidative biomarkers described below.

Serum superoxide dismutase (SOD) activities were evaluated spectrophotometrically as described by researchers [20], based on the superoxide anion inhibition reaction in the presence of pyragolol. Enzymatic activity was expressed in SOD units per mg of protein.

Serum GST activity was measured according to the literature [21] with minor modifications. GST activity was measured using the rate of formation of dinitrophenyl-S-glutathione at 340 nm in a medium containing 50 mM potassium phosphate, pH 6.5, 1 mM GSH, 1 mM 1-chloro-2,4-dinitrobenzene (CDNB) as substrate, and tissue supernatants (approximately 0.045 mg protein). Results were calculated and expressed as U per mg of protein.

Levels of TBARS (thiobarbituric acid reactive substances) were assessed to determine lipid peroxidation levels using the method described by Jentzsch et al. [22]. The TBARS results were obtained using a spectrophotometer at 535 nm and expressed as nmol MDA per mL.

Carbonines were measured using the reaction method of dinitrophenylhydrazine (DNPH) with protein carbonyls forming hydrazones, which are measured spectrophotometrically at 360 nm. For each sample, another tube was used, where the total protein of the sample was measured using HCl instead of DNPH; 20% TCA and then 0% TCA were added to both tubes to precipitate the carbonyls and proteins; the precipitate was washed with ethanol and ethyl acetate to remove free DNPH and contaminating lipids. The final precipitate was dissolved in guanidine and read at 360 nm, while protein concentration was read at 280 nm. Results were expressed in nM/mg protein.

### 2.9. Biomarkers in Rumen Liquid

The rumen fluid was collected four hours after feeding using a 1.5 m long, 11 mm diameter ororuminal probe and was allocated to 100 mL universal collectors. Part of the remaining rumen fluid was filtered through three gauzes, stored in 3 mL microtubes (Eppendorf^®^, Hamburg, Germany), and frozen at −20 °C for subsequent analysis of volatile fatty acids (VFAs).

To determine short-chain fatty acids (SCFAs), the fluid samples were thawed at 5 °C and manually shaken for homogenization. Aliquots of 1 mL of the ruminal fluid supernatant were collected into polypropylene microtubes (2 mL) and centrifuged for five minutes (12,300× *g*). Then, 250 μL of the supernatant was transferred to a new microtube containing 250 μL of formic acid. The mixture was manually shaken and centrifuged for three minutes. After centrifugation, 250 μL of the supernatant of the mixture was collected in another polypropylene tube previously containing 500 μL of 3-octanol solution (665 μg mL^−1^ in methanol) used as an internal standard. The mixture was homogenized and centrifuged again. Then, 600 μL of the sample was inserted into an injection vial and injected into a gas chromatograph equipped with a flame ionization detector (GC-FID; Varian Star 3400, Palo Alto, CA, USA) and an autosampler (Varian 8200CX, Palo Alto, CA, USA). One microliter of the extract was injected in split mode at 1:10. The carrier gas used was hydrogen at a constant pressure of 20 psi. The analytes (acetic, propionic, butyric, valeric, and isovaleric acids) were separated using a CP-Wax 52CB capillary column (60 m × 0.25 mm; 0.25 μm stationary phase thickness). The initial column temperature was set at 80 °C for one minute and increased to 120 °C at a rate of 8 °C min^−1^, then to 230 °C at 20 °C min^−1^, where it remained for one min. The injector and detector temperatures were set at 250 °C.

Method validation comprised the following parameters: selectivity, linearity, linear range, repeatability, precision, limit of detection (LOD), and limit of quantification (LOQ) for acetic, propionic, butyric, and isovaleric acids (Supplementary Materials S2). Linearity was assessed by calculating a regression equation using the least squares method. LOD and LOQ values were obtained by performing sequential dilutions until signal-to-noise ratios of 3:1 and 6:1 were achieved, respectively. Accuracy was assessed by analyzing the repeatability of six replicate samples. Accuracy was determined by recovering known amounts of standard substances added to a diluted sample. Results were expressed as mol 100 mol^−1^ of each SCFA in ruminal fluid.

### 2.10. Milk

#### 2.10.1. Composition and Quality

Analyses of chemical composition, standard plate count of bacteria, and SCC were carried out by the State Milk Quality Laboratory—LABLEITE of Concórdia—SC, which is accredited to the Ministry of Agriculture, Livestock and Supply by the MAPA ordinance: No. 212 of 31 July 2014. The quantification of fat, protein, lactose, total solids, and the defatted dry extract was quantified using the Mid-Infrared Spectrometry Method, according to ISO [23]. The Total Bacterial Count was carried out using the Flow Cytometry Method, according to ISO [24], and the SCC was measured using the Flow Cytometry Method, according to ISO [25].

#### 2.10.2. Fatty Acid Profile

To determine the fatty acid profile in milk, lipid extraction was performed using a specific method [26], but with adaptations; 1.5 g of samples, 0.8 mL of water, 5 mL of methanol, and 2.5 mL of chloroform were added to a 15 mL polypropylene tube, and mechanical stirring was carried out for 30 min. Next, 2.5 mL of chloroform solution and 1.5% NaSO_4_ were added to promote a two-phase system. This mixture was stirred for 2 min and then centrifuged for 15 min at 2000 rpm. The lipids obtained from the chloroform phase were subjected to fatty acid analysis.

FA methylation was performed using a transesterification method proposed by researchers [27]. One milliliter of 0.4 M KOH methanolic solution was added to the extracted lipids in a test tube and vortexed for one minute. The samples were kept in a water bath for ten minutes at boiling temperature. Subsequently, they were cooled to room temperature, and 3 mL of 1 M methanolic H_2_SO_4_ solution was added and vortexed for chromatographic analysis.

A gas chromatograph model TRACE 1310 with a flame ionization detector (Thermo Scientific, Milan, Italy) was used to determine FAME. One microliter of the sample was injected into a split/splitless injector, operated in split mode with a 1:20 ratio at 250 °C. Hydrogen was used as the carrier gas at a constant flow rate of 1.5 mL/min. Separation of FAMEs was performed using an RT 2560 chromatography column (100 m × 0.25 mm × 0.20 μm film thickness, Restek, Bellefonte, PA, USA). The initial oven temperature was programmed at 100 °C for five min and increased to 180 °C at 8 °C/min, followed by an increase to 210 °C at a rate of 4 °C/min, and finally up to 250 °C at 20 °C/min, maintaining it for 7 min under isothermal conditions. The detector temperature was kept constant at 250 °C. FAME compounds were identified by comparing the experimental retention time with those of the authentic standard (FAME Mix-37, Sigma Aldrich, St. Louis, MO, USA). Results were presented as a percentage of each FA identified in the lipid fraction, considering the factor equivalent to the size of the FAME chain for FID and the ester conversion factor for the respective acid, according to the literature [28]. Fatty acid profile results in TMR are presented in Supplementary Materials S3.

#### 2.10.3. Milk Microbiota

On days 30 (pool all cows—also day 0 of the experiment), 75, and 135 of lactation, milk was collected and stored in 3M™ Quick Swabs for transport to the laboratory, which reduced the qualitative and quantitative detection of microorganisms using metagenomics by sequencing the 16S rRNA gene, as performed by the laboratory BPI—Biotechnology Research and Innovation^®^, Sao Paulo, Brazil.

Total DNA was extracted from 200 mg (wet weight) of samples with the ZR Fungal/Bacterial DNA MiniPrep kit (Zymo Research, Tustin, CA, USA). Primers 515F (5′-GTGYCAGCMGCCGCGGTAA-3′) and 806R (5′-GGACTACNNGGGTATCTAAT-3′) were selected to amplify the V4 region of the bacterial 16S rRNA gene via the polymerase chain reaction [29].

Libraries were quantified using qualitative polymerase chain reaction with the Kapa Library Quantification Kit (Illumina, San Diego, CA, USA), following the manufacturer’s recommendations. Samples were normalized to a final concentration of 2 nM and sequenced with an Illumina MiSeq for 250 cycles from each end.

### 2.11. Apparent Digestibility Coefficients

Researchers described using indigestible neutral detergent fiber (iNDF) to determine apparent digestibility [30]. Feed and feces samples were incubated in bovine rumen for 288 h, then washed and dried in a forced ventilation oven. NDF and ADF concentrations were determined to calculate digestibility [31].

### 2.12. Statistical Analysis

Data were tested for normality and homogeneity of variance using the Shapiro–Wilk and Levene tests, respectively. All data were analyzed using the SAS “MIXED procedure” (SAS Inst. Inc., Cary, NC, USA; version 9.4). The Satterthwaite approximation was used to determine the denominator degrees of freedom for the fixed effects test. Feed efficiency was tested for the fixed effect of treatment using animal (treatment) as a random effect. The remaining data were analyzed as repeated measures and tested for fixed effects of treatment and treatment × day, using animal (treatment) as a random effect. Day-one results were included as an independent covariate. Means were separated using the PDIFF method (Student’s test), and all results were reported as LSMEANS followed by SEM. Significance was defined when *p* ≤ 0.05, and trend when *p* > 0.05 and ≤0.10.

Sequence data were processed using Mothur v.1.39.5 [32], which aligns with the Mothur MiSeq SOP [33]. Taxonomy was assigned by querying the representative sequence of each oligotype against the SILVA database (release 132) [34] and Greengenes [35]. Closed-reference clustered operational taxonomic unit data were exported for analysis with Phyloseq v1.41 [36] in R 4.3.1 (R Core Team, 2023). The Permanova test was used to compare the abundance of specific taxons between groups. Taxonomic alpha diversity was estimated using Chao1 richness and the Shannon, Simpson, and Fisher indices, which account for both richness and evenness. Beta diversity was assessed using the Bray–Curtis distance, and taxonomic dissimilarity among samples was explored using principal coordinates analysis. Differential abundance of taxa was calculated using a negative Binomial method implemented in the DESeq2 package [37] to identify individual virtual taxa that were more or less abundant when comparing phytobiotic or control conditions. All figures were generated using the ggplot2 package v3.2.1 [38].

## 3. Results

### 3.1. Milk Production, Feed Intake and Feed Efficiency

Data on milk production, dry matter intake (DMI), and feed efficiency from early lactation and mid-lactation are shown in Table 2. Milk production was not affected by the treatment in the two lactation phases. However, a treatment × day interaction was detected in both production moments, sometimes more significant in cows that consumed the additive (Supplementary Materials S4). There was no effect of treatment and treatment × day interaction on DMI in early lactation and mid-lactation. The feed efficiency of cows in the phytogenic group was higher compared to the control group during early lactation. At the same time, in mid-lactation, there was only a trend toward greater feed efficiency in the treated animals compared to the control.

### 3.2. Hematologic Changes

The cows’ blood count results are described in Table 3. During early lactation or mid-lactation, there was no effect of treatment and treatment × day interaction on the variables of erythrocytes, hemoglobin, hematocrit, granulocytes, monocytes, and platelets. Treatment × day interaction was also not verified for the WBC variables. Nevertheless, during the peak lactation period (early lactation), there was an effect of treatment on the total leukocyte count, with the number of cells being lower in animals in the treatment group; this effect was related to a tendency for lower lymphocyte counts in these cows compared to the control. In mid-lactation, there was a tendency toward a treatment effect on the total leukocyte count (lower in cows that consumed the additive), which was due to an effect of treatment on lymphocyte count, as the number of cells was lower in cows in the treatment group compared to the control. There was no treatment effect on the granulocyte and monocyte variables between treatments.

### 3.3. Serum Biochemistry, Proteinogram, and Oxidative Status

The serum biochemistry results are shown in Table 4. The groups had no treatment effect or treatment × day interaction for albumin, fructosamine, CRP, total protein, and urea variables. In the first period of mechanical milking (early lactation), treatment affected cholesterol levels, which was lower in cows in the phytogenic group compared to the control group. At the same time, there was no difference between treatments in mid-lactation. In early lactation, treatment affected the GGT enzyme, with its activity being lower in cows in the phytogenic group compared to the control; however, this effect was not verified in mid-lactation. In early lactation, there was no effect of treatment and no treatment × day interaction for AST activity. Still, in mid-lactation, AST activity impacted the treatment, being lower in the serum of cows in the phytogenic group compared to the control. There was no treatment × day interaction for globulins, but there was an effect of the treatment; that is, in early lactation, higher levels of globulins were identified in cows in the phytogenic group compared to the control, different from what occurred in mid-lactation, when the level of globulins tended to be lower in the serum of cows in the phytogenic group.

The protein profile values from electrophoresis are described in Table 5. There was no effect of treatment and treatment × day interaction on the concentration of heavy chain immunoglobulins, transferrin, ferritin, and serum amyloid during the two periods of the experiment. There was an effect of treatment and treatment × day interaction (DL 45 and 60) for IgA levels in the period I of the experiment, being higher in the serum of cows that consumed the additive compared to the control (*p* ≤ 0.05); however, this effect was not verified in mid-lactation (Supplementary Materials S5). In early lactation and mid-lactation phases, there was an effect of treatment (Table 5) and treatment × day interaction for ceruloplasmin and haptoglobin levels measured at lower levels in cows that consumed the additive compared to the control (Supplementary Materials S5).

The results for oxidative and antioxidant biomarkers are presented in Table 6. Only during early lactation was there an effect of treatment and treatment × day interaction on SOD activity, which was lower in the serum of animals in the phytogenic group compared to the control (Supplementary Materials S6). GST activity was affected by treatment and interaction in both lactation phases (Supplementary Materials S6); the serum activity was more significant when the cows consumed the phytogenic additive. There was an effect of treatment (Table 6) and treatment × day interaction on TBARS concentration in both research moments (Supplementary Materials S6), with lower levels in cows from the phytogenic group. For carbolines, only treatment × day interaction was observed in early lactation, with the concentration being lower in animals in the phytogenic group (Supplementary Materials S6).

### 3.4. Ruminal Environment

The results of the profile of VFA in the ruminal environment are described in Table 7. The effect of treatment was observed for SCFA levels, which were higher in animals in the phytogenic group at DIM 75 and 135. The impact of treatment on acetic acid proportion was observed only at DIM 135 when higher percentages were found in cows in the phytogenic group compared to the control. The effect of treatment on butyric acid was lower at both moments (DIM 75 and 135) in the ruminal fluid of cows that consumed the phytogenic additive compared to the control. The effect of treatment on the concentration of valeric acid was observed on days 75 and 135, being lower in cows that consumed the additive compared to the control. The acetic acid/propionic acid ratio tended to be higher in the phytogenic group compared to the control. There was no treatment effect on propionic acid and isovaleric acid percentages between treatments in this experiment.

### 3.5. Apparent Digestibility Coefficient

Table 8 describes the results of the apparent digestibility coefficient (ADC). During the mechanical milking period (early lactation), the treatment had no effect on the ADC of DM and nutrients (OM, CP, NDF, ADF, and EE). In the robotic milking period (mid-lactation), the treatment had an impact, with a higher ADC for MS, MO, PB, NDF, FDA, and EE in cows that consumed the additive compared to the control.

### 3.6. Milk Analyses

Table 9 presents the results of the milk composition. No treatment effect was observed for the percentage of fat, protein, lactose, total solids, standard plate count, defatted dry extract, and casein between experimental groups. In early lactation, the treatment had no effect; however, in two lactation phases, there was a tendency for a treatment × day interaction for SCC, with SCC being lower in the milk of cows in the phytogenic group. In mid-lactation, the effect of the treatment on SCC was smaller in the phytogenic group (*p* > 0.05).

In this early lactation, a trend toward a treatment effect was observed for the concentration of urea in milk (*p* < 0.05–>0.10), being higher in the milk of cows in the phytogenic group. In mid-lactation, the urea concentration was significantly higher in the milk of cows that consumed the additive.

Table 10 describes the results of the milk fatty acid profile in early lactation (mechanical milking). An effect of treatment and treatment × day interaction was observed, with lower percentages of palmitic acid and henicosanoic acid being measured in the cows’ milk in the phytogenic group (*p* > 0.05). There was an effect of treatment and a treatment × day interaction for oleic acid, where a higher concentration of oleic acid was found in the milk of cows that consumed the additive compared to the control group. For the sum of acids, there was an effect of treatment for saturated fat (lower in milk from the phytogenic group) and a treatment × day interaction (DIM 60), which was lower in the phytogenic group compared to the control. While the sum of unsaturated and monounsaturated fats was higher in the phytogenic group, there was also a treatment × day interaction, with higher levels at DIM 60. The unsaturated/saturated fat ratio was affected by treatment and a treatment × day interaction (DIM 60 and 75), which was higher in the cows that consumed the additive. There was no effect on treatment and treatment interaction for the other fatty acids presented in Supplementary Materials S7.

The results of the mid-lactation of the experiment are described in Table 10. A treatment × day interaction was verified for palmitic and oleic acid (*p* > 0.05), with palmitic acid being lower and oleic acid being higher in the cows that consumed the additive at DIM 135. The sum of saturated and unsaturated fatty acids did not differ between treatments, as the other fatty acids detected in chromatography and presented in Supplementary Materials S7.

**Figure 1:**
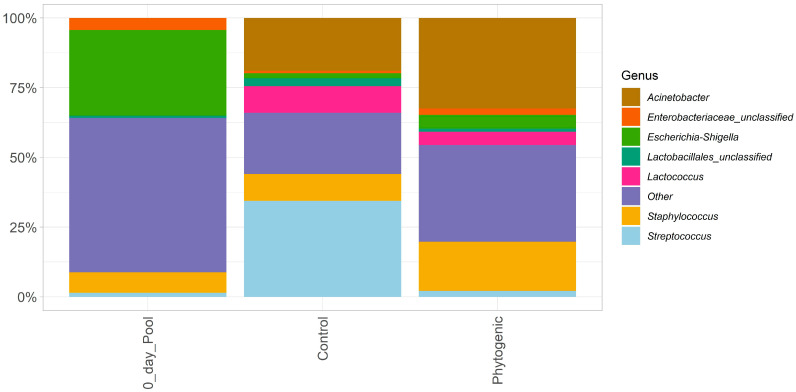
Percentage abundance of microorganisms present in cows’ milk before the beginning of the experiment (pool of all cows on day 0 of the experiment = DIM 30) and at the end of each experimental stage (DIM 75 and DIM 135). Note: phytogenic group: animals’ intake of an additive formulated with a combination of cinnamon and oregano essential oil, chelated amino acid chromium, selenium proteinate, inactivated *Saccharomyces cerevisiae*, *S. cerevisiae*, turmeric extract, and tannic acid.

Figure 1 presents the results of microbiota abundance in cows’ milk. We found that the primary bacteria were identified as *Acinetobacter* spp., *Escherichia-Shigella* spp., *Lactococcus* spp., *Staphylococcus* spp., and *Streptococcus* spp., as well as the family (*Enterobacteriaceae*) and order (*Lactobacillales*).

**Figure 2:**
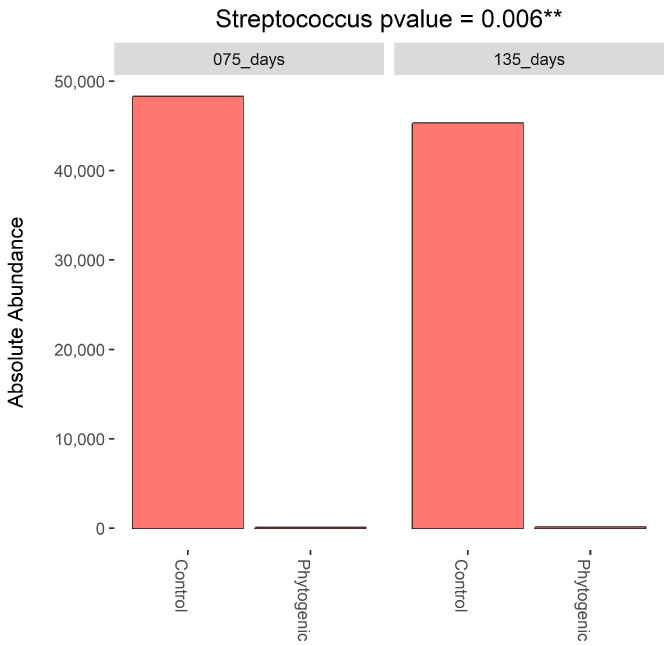
Several sequences of *Streptococcus* were observed in milk at 75 (peak lactation stage) and 135 (middle lactation stage) days of lactation. Note: phytogenic group: animals’ intake of an additive formulated with a combination of cinnamon and oregano essential oil, chelated amino acid chromium, selenium proteinate, inactivated *Saccharomyces cerevisiae*, *S. cerevisiae*, turmeric extract, and tannic acid. Marked with two (**) if *p*-value < 0.01.

**Figure 3:**
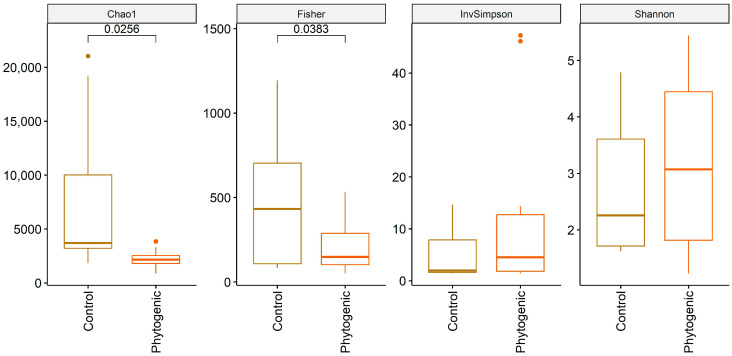
Alpha diversity of microorganisms in milk from dairy cows during early lactation (30–75 DIM) and mid-lactation (90–135 DIM) assessed using three data analysis models (Chao1, Fisher, InvSimpson and Shannon): control (CON) vs. phytogenic (PHY). Note: phytogenic group: animals’ intake of an additive formulated with a combination of cinnamon and oregano essential oil, chelated amino acid chromium, selenium proteinate, inactivated *Saccharomyces cerevisiae*, *S. cerevisiae*, turmeric extract, and tannic acid. If there are values that fall above or below the end of the whiskers, they are plotted as dots.

Statistically, a lower abundance of *Streptococcus* spp. was found in the milk of cows that consumed the phytogenic additive (Figure 2). Alpha diversity results (Figure 3) showed that the richness indices (Chao1 and Fisher) were significant, while the evenness indices (InvSimpson and Shannon) were not statistically different. Beta diversity (Supplementary Materials S8) demonstrated individual variability between animals, regardless of treatment, as seen in the seven most abundant taxa presented individually at both moments (DIM 75 and 135).

## 4. Discussion

The sum of milk produced by cows that consumed the phytogenic product was higher, but not significantly higher, during the two periods. However, at some points in the study, production was higher in the cows that consumed the additive. In early lactation, we found that after 25 days of ingesting the additive, the milk production of cows in the phytogenic group was higher. In mid-lactation, we observed a drop in the productivity of animals in both groups, a factor related to the change in the milking system, as already demonstrated by other researchers [39,40,41]. However, after the adaptation period of this mid-lactation, the cows that consumed phytogenics produced more milk daily compared to the control group at various times, notably during the last seven days of the experiment. However, the change in the milking system (from mechanical to robotic) influenced the productivity of cows, as they needed to adapt to the new one (robotic milking system), and thus during the adaptation period and beginning of lactation in the second phase of this study, we observed no difference in milk production between experimental groups. This is a possible limitation of the effect of the tested additive due to interference from the milking management. Despite this, the treatment did not affect feed consumption, but feed efficiency was more significant in cows that consumed phytogenics. At the peak of lactation, greater milk production is related to better cow health, as the feed additive, when consumed, stimulates an immune, anti-inflammatory, and antioxidant response. Already in the mid-lactation phase, in addition to these serum results, productivity improvements may result from the more significant amount of VFAs in the ruminal liquid and the greater digestibility of nutrients when consuming the additive. Therefore, it is clear that phytogenics directly affects nutrition, providing better absorption of nutrients.

The more significant amount of VFA in the cows’ ruminal environment is a consequence of higher concentrations of acetic acid and valeric acid in the animals that consumed the phytogenic mixture. These results lead us to understand that the additive was capable of modulating the rumen environment, just as it tended to increase the acetate/propionate ratio. This is the first study with this phytogenic combination of essential oils (free and encapsulated), plant extracts, minerals, and microorganisms. Still, the isolated nutritional effects of these blend ingredients are already well known. Using oregano and cinnamon essential oil changes the acetate/propionate ratio and the amount of VFA [42]. In an in vitro study carried out in [43], when essential oils were used, there was a tendency to increase VFAs, as well as when using a blend of essential oils (oregano and cinnamon) in the diet of sheep [44], they had changes in rumen fermentation and increased milk production. Using a blend of phytogenics with ingredients similar to those in this study, authors [45] found an increase in acetic acid in Holstein steers. Researchers [46] also described an increase in acetic acid in dairy cows when using *Saccharomyces cerevisiae*, which is another additive present in our blend. Researchers found a stimulation of nitrogen retention induced by curcumin in cattle, which was considered a potentially positive effect. However, a decrease in the apparent digestibility of ADF may limit forage utilization [47], unlike a study with dairy sheep with greater NDF digestibility [9]. Tannic acid supplementation in cattle reduced the percentages of butyrate, isobutyrate, and valerate; however, it increased the proportion of acetate and the acetate/propionate ratio [48], which demonstrates that tannins alter the profile of VFA in the rumen environment, similar to what was observed in this study with the feed additive.

VFA is directly linked to the digestibility of feed; in our study, it was found that digestibility had a positive effect on the phytogenic group only at the end of the experiment (after consuming the additive for more than 100 days), with higher digestibility coefficients observed for dry matter, organic matter, protein, fiber, and fat. However, it is essential to highlight that the diets during early lactation and mid-lactation differed due to the inclusion of concentrate available in the robot during milking. This difference in TMR between the two phases is probably the reason for increasing the digestibility coefficients when the cows were in a stable condition (mid-lactation), different from a cow at peak production, and still under the effects of metabolic disorders arising from the transition phase. However, these results on digestibility need to be further elucidated. Similar effects have already been described in sheep that used curcumin, where it had greater digestibility of DM and NDF [9,49]. Cinnamon essential oil in cattle diets increased the digestion of OM [50], and the addition of *Saccharomyces cerevisiae* to the diets of sheep and goats increased the digestibility of DM, OM, CP, NDF, and ADF [51,52]. Researchers [53] used a mixture of phytogenic additives in dairy cows and obtained results similar to ours, with an increase in nutrient digestibility. Therefore, it is suggestive that in phase 2 of this experiment (mid-lactation), the additive ingredients used here have high potential in animal nutrition and are nutritionally efficient in enhancing animal production; however, in phase 1 (peak lactation), it is believed that productivity was higher in cows that consumed the additive as an indirect effect, that is, it improved the cow’s sweat, reducing energy expenditure on inflammation and oxidative reactions.

We observed a lower leukocyte count due to fewer lymphocytes in cows that consumed the additive. These variables can be used as markers of stress [54] and inflammatory processes [55,56]. This reduction in white blood cells was also observed when curcumin was used in dairy sheep [12] and dairy calves [57], as well as when using microencapsulated cinnamon and oregano oil in lambs [58]. Still related to the hypothesis that the additive provides an anti-inflammatory response, we had lower concentrations of acute phase proteins such as ceruloplasmin and haptoglobin, which are biomarkers known as indicators of a pro-inflammatory process [59,60]. This is likely related to the anti-inflammatory action of turmeric [61], cinnamon, and oregano essential oil, which has anti-inflammatory properties [62,63]. All the factors mentioned above are directly related to the lower SCC in milk presented by cows in the phytogenic group in our study, showing that when the ingredients are used together, they do not lose their effects. It is important to highlight that turmeric extract and cinnamon and oregano essential oils are some of the ingredients used in the production of the feed additive supplied to cows, as well as probiotics and minerals (selenium and chromium), also well-known as immune response stimulators [64,65]. In addition to reducing inflammation, the feed additive stimulated the IgA immune response, similar to the study by authors [45], when a blend of phytogenics similar to the additive used here was used. The combination of these results related to the immune system was beneficial to the cow’s health and, consequently, to milk production, combined with the nutritional effects already discussed.

The oxidative biomarkers were influenced by the consumption of the feed additive by the cows, highlighting lower SOD activity and lower TBARS levels, which shows the antioxidant potential of the phytogenic mixture. Antioxidant actions are found in animals that consumed curcumin [66], selenium, and chromium [64,65,67] and highlighted that cinnamon and oregano essential oils have components with the capacity to absorb oxygen radicals [68], which may justify the lower lipid peroxidation observed here. GST activity was higher in the group of cows that consumed the phytogenic mixture because the additive has a liver detoxification effect, and this antioxidant enzyme is the most important in this tissue and localized detoxification process. This effect is similar to that found when using curcumin in sheep [12] and a phytogenic blend in steers [45]. Therefore, the combination of ingredients in the production of the phytogenic mixture was efficient in stimulating the antioxidant response and consequently reducing oxidative reactions, which, when exacerbated, harmed the cow’s health and interfered with milk production. Therefore, this desirable antioxidant effect may have indirectly contributed to enhancing milk production and reducing SCC in the milk.

The fatty acid profile of milk is entirely linked to the cow’s diet and, therefore, can vary according to the feed consumed [69]. In our study, we observed a lower concentration of palmitic and heneicosanoic acids in the milk of cows that consumed the phytoactive mixture. Consequently, there was a lower amount of saturated fatty acids. We assessed that cows that consumed the phytogenic mixture had a higher proportion of oleic acid, contributing to a more significant sum of monounsaturated fatty acids. When using curcumin in dairy sheep, similar results were observed: lower presence of palmitic acid in the treated group and a more significant treatment effect for oleic acid and monounsaturated fatty acids [9]. The use of tannins in goats also resulted in an increase in oleic acid [70]. These results show that using additives is a way to positively modulate milk quality to have significant amounts of omegas in the milk and lower somatic cell counts. Ingesting feeds with lower amounts of saturated fatty acids is a recommendation from nutritionists, as saturated fat can deposit in blood vessels and, over time, cause cardiovascular problems [71].

The consumption of the phytogenic additive by cows affected the abundance of *Streptococcus spp*. in milk, reducing its occurrence. Furthermore, our research identified the genera *Acinetobacter* spp., *Escherichia-Shigella* spp., and *Lactococcus* spp. as the main microorganisms in milk. Moreover, *Streptococcus* spp. is a genus of bacteria commonly responsible for causing mastitis [72,73,74]. In in vitro studies, cinnamon essential oil has demonstrated antibiofilm potential and inhibitory activity against bacteria of the genus *Streptococcus* spp. [75]. It also disrupts the membrane’s integrity and stops pathogens’ ATP production [76]. According to [77], curcumin associated with hydrogen peroxide has shown an antimicrobial effect against *Streptococcus* spp. In a recent study, cows were infected with *Streptococcus uberis* in a mammary quarter and fed with *Saccharomyces cerevisiae* in their diet; this resulted in lower SCC and temperature of the infected quarter; further, this resulted in lower CCS and infected room temperature—furthermore, the prebiotic improved mammary genes related to the antibacterial function of cells [78]. As observed in our study, these phytogenics could inhibit or recover animals from problems caused by *Streptococcus* spp.

## 5. Conclusions

The results allow us to conclude that adding the phytoactive mixture to the dairy cows’ diet positively affected animal nutrition and health, improving the production efficiency of Jersey cows. We also concluded that the intake of phytogenics improved milk quality, reduced SCC, increased unsaturated fatty acids in milk, and protected the mammary gland from the bacteria *Streptococcus* spp. It is believed that consumption of the phytoactive mixture during mid-lactation had direct effects on digestibility and the modulation of the volatile fatty acid profile while also having an indirect impact by stimulating an immune and antioxidant response combined with anti-inflammatory action in two experimental phases.

## Figures and Tables

**Table 1:** Chemical composition of feed and total diet fed to dairy cows during early lactation (30–75 DIM) and mid-lactation (90–135 DIM).

	Silage	Basal Concentrate	TMR Control	TMR Phytogenic	Robot Feed—Pelleted
Early lactation: DIM 30–75					
Dry matter	27.35	86.06	48.6	48.81	-
Crude protein	10.56	26.33	16.83	16.86	-
Ethereal extract	2.45	6.79	3.77	3.83	-
Ash	5.91	8.84	9.26	8.71	-
NDF	57.85	29.77	43.75	43.5	-
ADF	26.43	17.21	22.29	21.36	-
Mid-lactation: DIM 90–135					
Dry matter	25.09	84.07	43.8	41.8	88.75
Crude protein	9.88	26.15	16.8	15.8	20.87
Ethereal extract	2.9	2.77	2.69	3.32	5.51
Ash	3.74	7.6	9.17	8.49	10.74
NDF	40.35	22.5	39.24	40.35	30.06
ADF	19.62	12.82	19.17	20.5	13.86

Concentrate (Soybean meal, corn, soybean hulls, wheat bran, mineral, and sodium bicarbonate); TMR: in the total mixed ration; NDF: neutral detergent fiber; ADF: acid detergent fiber; DIM: days in milk.

**Table 2:** Productive performance of dairy cows during early lactation (30–75 DIM) and mid-lactation (90–135 DIM).

Exp. Period	Group	Production (kg) of Milk/Cow/Day	Consumption (kg) Feed/Cow/Day	Feed Efficiency
Early lactation				
DIM 30 to 75	CON	23.3	14.34	1.62 ^b^
	PHY ^1^	24.8	14.01	1.75 ^a^
	SEM	0.48	0.19	0.03
	P treat	0.25	0.82	0.05
	P treat × day	0.01	0.74	-
Mid-lactation				
DIM 90 to 135	CON	19.7	15.17	1.29 ^b^
	PHY ^1^	20.8	15.21	1.36 ^a^
	SEM	0.42	0.18	0.07
	P treat	0.52	0.92	0.09
	P treat × day	0.05	0.95	-

Note: ^a,b^ Significance differences between groups (column) were defined when *p* ≤ 0.05, and trend when *p* > 0.05 and ≤0.10; DIM: days in milk; kg: kilograms. ^1^ The phytogenic additive is a combination of cinnamon and oregano essential oil, chelated amino acid chromium, selenium proteinate, inactivated *Saccharomyces cerevisiae*, *S. cerevisiae*, turmeric extract, and tannic acid.

**Table 3:** Blood count of dairy cows during early lactation (30–75 DIM) and mid-lactation (90–135 DIM).

Exp. Period	Group	ERY	HEM	HT	LEU	LYM	GRA	MON	PLA
Early lactation									
DIM 30 to 75	CON	5.63	9.56	26.6	5.34 ^a^	3.00 ^a^	1.49	1.10	443
	PHY ^1^	5.12	8.88	25.5	4.29 ^b^	2.40 ^b^	1.15	0.88	391
	SEM	0.14	0.18	0.38	0.32	0.16	0.09	0.08	27.9
	P treat	0.38	0.45	0.64	0.05	0.09	0.21	0.48	0.55
	P treat × day	0.24	0.32	0.41	0.11	0.17	0.12	0.56	0.31
Mid-lactation									
DIM 90 to 135	CON	5.85	9.88	27.7	6.30 ^a^	3.44 ^a^	1.64	1.22	318
	PHY ^1^	5.51	9.79	27.1	5.28 ^b^	2.97 ^b^	1.33	0.99	255
	SEM	0.16	0.29	0.69	0.34	0.13	0.09	0.06	27.6
	P treat	0.87	0.93	0.95	0.07	0.05	0.52	0.74	0.40
	P treat × day	0.92	0.90	0.96	0.16	0.12	0.20	0.53	0.21

Note: ^a,b^ Significance differences between groups (column) were defined when *p* ≤ 0.05, and trends when *p* > 0.05 and ≤0.10. DIM: days in milk; ERY: erythrocytes (×10^6^ µL); HEM: hemoglobin (mg/dL); HT: hematocrit (%); LEU: leukocytes (×10^3^ µL); LYM: lymphocyte (×10^3^ µL); GRA: granulocyte (×10^3^ µL); MON: monocyte (×10^3^ µL); PLA: platelets (×10^3^ µL). ^1^ The phytogenic additive is a combination of cinnamon and oregano essential oil, chelated amino acid chromium, selenium proteinate, inactivated *Saccharomyces cerevisiae*, *S. cerevisiae*, turmeric extract, and tannic acid.

**Table 4:** Serum biochemistry of dairy cows during early lactation (30–75 DIM) and mid-lactation (90–135 DIM).

Exp. Period	Group	ALB	CHO	FRU	GGT	CRP	TP	AST	URE	GLO
Early lactation										
DIM 30 to 75	CON	3.25	163 ^a^	239	28.5 ^a^	2.96	7.39	85.9	50.0	4.14 ^b^
	PHY ^1^	3.16	144 ^b^	236	23.8 ^b^	2.84	7.78	96.6	52.9	4.62 ^a^
	SEM	0.06	5.40	5.02	1.53	0.03	0.08	3.07	1.43	0.05
	P treat	0.68	0.03	0.91	0.05	0.46	0.69	0.24	0.46	0.05
	P treat × day	0.84	0.15	0.97	0.10	0.65	0.75	0.22	0.32	0.13
Mid-lactation										
DIM 90 to 135	CON	3.24	149	239	33.4	2.97	7.51	108 ^a^	45.7	4.27 ^a^
	PHY ^1^	3.31	155	235	32.5	2.97	7.27	97.3 ^b^	48.4	3.96 ^b^
	SEM	0.02	2.85	3.00	3.01	0.03	0.06	3.48	0.94	0.06
	P treat	0.85	0.74	0.95	0.88	0.97	0.89	0.09	0.39	0.08
	P treat × day	0.89	0.62	0.92	0.91	0.94	0.91	0.12	0.24	0.37

^a,b^ Significance differences between groups (column) were defined when *p* ≤ 0.05, and trends when *p* > 0.05 and ≤0.10. DIM: days in Milk; ALB: albumin (g/dL), CHO: cholesterol (mg/dL), FRU: fructosamine (µmol/L); GGT: gamma-glutamyl transferase (U/L), reactive-C protein (CRP) (g/dL), TP: total protein (g/dL), AST: aspartate aminotransferase (U/L), URE: urea (mg/dL), GLO: globulin (g/dL). ^1^ The phytogenic additive is a combination of cinnamon and oregano essential oil, chelated amino acid chromium, selenium proteinate, inactivated *Saccharomyces cerevisiae*, *S. cerevisiae*, turmeric extract, and tannic acid.

**Table 5:** Serum proteinogram of dairy cows during early lactation (30–75 DIM) and mid-lactation (90–135 DIM): control (CON) vs. phytogenic (PHY).

Exp. Period	Group	IgA	Ig-HC	CERU	TRAN	FER	HAPT	SE-A
Early lactation								
DIM 30 to 75	CON	0.78 ^b^	0.99	0.76 ^a^	0.35	0.42	0.25 ^a^	0.21
	PHY ^1^	0.84 ^a^	1.07	0.45 ^b^	0.33	0.42	0.19 ^b^	0.20
	SEM	0.02	0.04	0.02	0.02	0.03	0.02	0.01
	P treat	0.05	0.35	0.01	0.65	0.95	0.01	0.92
	P treat × day	0.02	0.12	0.01	0.73	0.90	0.01	0.97
Mid-lactation								
DIM 90 to 135	CON	0.77	0.94	0.80 ^a^	0.29	0.41	0.31 ^a^	0.23
	PHY ^1^	0.81	0.98	0.52 ^b^	0.31	0.37	0.20 ^b^	0.25
	SEM	0.02	0.04	0.02	0.02	0.04	0.01	0.02
	P treat	0.19	0.82	0.01	0.52	0.13	0.01	0.77
	P treat × day	0.13	0.89	0.01	0.63	0.24	0.01	0.55

^a,b^ Significance differences between groups (column) were defined when *p* ≤ 0.05, and trends when *p* > 0.05 and ≤0.10. DIM: days in Milk; IgA: immunoglobulin A (g/dL), Ig-HC: Ig heavy chain (g/dL), CERU: ceruloplasmin (g/dL), TRAN: transferrin (g/dL); FER: ferritin (g/dL), HAPT: haptoglobin (g/dL), SE-A: serum amyloid (g/dL). ^1^ The phytogenic additive is a combination of cinnamon and oregano essential oil, chelated amino acid chromium, selenium proteinate, inactivated *Saccharomyces cerevisiae*, *S. cerevisiae*, turmeric extract, and tannic acid.

**Table 6:** Serum oxidative status of dairy cows during early lactation (30–75 DIM) and mid-lactation (90–135 DIM: control (CON) vs. phytogenic (PHY).

Exp. Period	Group	SOD	GST	TBARS	CAB-P
Early lactation					
DIM 30 to 75	CON	14.0 ^a^	16.8 ^b^	38.3 ^a^	5.15
	PHY ^1^	11.7 ^b^	23.1 ^a^	23.2 ^b^	4.19
	SEM	0.17	1.38	1.24	0.45
	P treat	0.04	0.03	0.01	0.24
	P treat × day	0.01	0.01	0.01	0.03
Mid-lactation					
DIM 90 to 135	CON	11.3	15.5 ^b^	20.9 ^a^	7.76
	PHY ^1^	11.5	22.7 ^a^	13.0 ^b^	7.64
	SEM	0.12	0.84	1.39	0.29
	P treat	0.89	0.03	0.05	0.91
	P treat × day	0.93	0.01	0.02	0.95

^a,b^ Significance differences between groups (column) were defined when *p* ≤ 0.05, and trends when *p* > 0.05 and ≤0.10. DIM: days in Milk; SOD: superoxide dismutase (U SOD/mg protein), GST: glutathione S-transferase (U GST/mg protein), TBARS: lipid peroxidation (nmol MDA/mL); CAB-P: carbonyl protein (nmol of carbonyl groups/mg of protein). ^1^ The phytogenic additive is a combination of cinnamon and oregano essential oil, chelated amino acid chromium, selenium proteinate, inactivated *Saccharomyces cerevisiae*, *S. cerevisiae*, turmeric extract, and tannic acid.

**Table 7:** Profile of short-chain fatty acids in the rumen liquid of cows in each experimental period (early lactation—DIM 75 and mid-lactation—DIM 135): control (CON) vs. phytogenic (PHY).

	CON	PHY ^1^	SEM	P: Treat
VFA, mmol/L				
DIM 75	84.18 ^b^	96.71 ^a^	0.43	0.01
DIM 135	72.68 ^b^	82.83 ^a^	0.41	0.01
Acetic acid (%)				
DIM 75	65.09	68.64	0.35	0.02
DIM 135	63.68 ^b^	69.85 ^a^	0.36	0.01
Propionic acid (%)				
DIM 75	16.60	15.48	0.14	0.67
DIM 135	16.5	15.51	0.13	0.62
Butyric acid (%)				
DIM 75	16.59 ^a^	13.47 ^b^	0.15	0.01
DIM 135	15.30 ^a^	12.00 ^b^	0.14	0.02
Isovaleric acid (%)				
DIM 75	1.44	1.31	0.12	0.52
DIM 135	1.42	1.33	0.12	0.58
Valeric acid (%)				
DIM 75	1.52 ^a^	1.15 ^b^	0.05	0.01
DIM 135	1.61 ^a^	1.33 ^b^	0.05	0.05
Acetic/propionic				
DIM 75	4.30	4.59	0.17	0.65
DIM 135	3.66 ^b^	5.00 ^a^	0.18	0.07

Note: ^a,b^ Significance differences between groups (line) were defined when *p* ≤ 0.05, and trends when *p* > 0.05 and ≤0.10. DIM: days in milk. ^1^ The phytogenic additive is a combination of cinnamon and oregano essential oil, chelated amino acid chromium, selenium proteinate, inactivated *Saccharomyces cerevisiae*, *S. cerevisiae*, turmeric extract, and tannic acid.

**Table 8:** Apparent nutrient digestibility coefficient of dairy cows during early lactation (30–75 DIM) and mid-lactation (90–135 DIM: control (CON) vs. phytogenic (PHY).

Exp. Period	Group	Dry Matter	Organic Matter	Crude Protein	NDF	ADF	EE
Early lactation							
DIM 73,74, 75	CON	0.68	0.71	0.68	0.59	0.57	0.83
	PHY ^1^	0.67	0.70	0.67	0.60	0.56	0.82
	SEM	0.01	0.01	0.02	0.02	0.02	0.01
	P treat	0.97	0.96	0.95	0.95	0.94	0.96
Mid-lactation							
DIM 133, 134, 135	CON	0.56 ^b^	0.60 ^b^	0.57 ^b^	0.51 ^b^	0.55 ^b^	0.65 ^b^
	PHY ^1^	0.71 ^a^	0.73 ^a^	0.74 ^a^	0.69 ^a^	0.73 ^a^	0.81 ^a^
	SEM	0.04	0.03	0.06	0.05	0.05	0.06
	P treat	0.01	0.01	0.01	0.01	0.01	0.01

^a,b^ Differs (*p* ≤ 0.05) between treatments (column). DIM: days in Milk; NDF: neutral detergent fiber; ADF: acid detergent fiber; EE: ethereal extract. ^1^ The phytogenic additive is a combination of cinnamon and oregano essential oil, chelated amino acid chromium, selenium proteinate, inactivated *Saccharomyces cerevisiae*, *S. cerevisiae*, turmeric extract, and tannic acid.

**Table 9:** Chemical composition in the milk of dairy cows during early lactation (30–75 DIM) and mid-lactation (90–135 DIM: control (CON) vs. phytogenic (PHY).

Exp. Period	Group	Fat (g/100 g)	Protein (g/100 g)	Lactose (g/100 g)	TS (g/100 g)	SCC (×1000/mL)	DDE (g/100 g)	Urea (mg/dL)	Casein (g/100 g)
Early lactation									
DIM 30 to 75	CON	4.47	3.27	4.66	13.3	68.2	8.84	17.0 ^b^	2.58
	PHY ^1^	4.10	3.24	4.62	12.9	50.8	8.78	19.3 ^a^	2.50
	SEM	0.28	0.04	0.03	0.27	8.64	0.05	0.61	0.04
	P treat	0.82	0.95	0.97	0.78	0.14	0.95	0.06	0.89
	P treat × day	0.71	0.92	0.98	0.86	0.07	0.91	0.17	0.93
Mid-lactation									
DIM 90 to 135	CON	2.96	3.56	4.70	12.2	72.5 ^a^	9.20	14.1 ^b^	2.85
	PHY ^1^	3.16	3.51	4.50	12.1	44.9 ^b^	8.94	16.7 ^a^	2.81
	SEM	0.21	0.04	0.03	0.23	12.5	0.06	0.41	0.04
	P treat	0.74	0.92	0.85	0.94	0.04	0.88	0.03	0.95
	P treat × day	0.81	0.95	0.91	0.9	0.09	0.81	0.11	0.95

^a,b^ Significance differences between groups (line) were defined when *p* ≤ 0.05, and trends when *p* > 0.05 and ≤0.10. DIM: days in Milk; ST: total solids; SCC: somatic cell count; DDE: defatted dry extract. ^1^ The pPhytogenic additive is a combination of cinnamon and oregano essential oil, chelated amino acid chromium, selenium proteinate, inactivated *Saccharomyces cerevisiae*, *S. cerevisiae*, turmeric extract, and tannic acid.

**Table 10:** Profile of fatty acids in milk from dairy cows during early lactation (30–75 DIM) and mid-lactation (90–135 DIM: control (CON) vs. phytogenic (PHY).

Fatty Acid	CON	PHY ^1^	SEM	P Treat	P Treat × Day
Early lactation					
C16:0 (Palmitic)				0.05	0.01
DIM 60	38.7 ^a^	34.6 ^b^	0.720		
DIM 75	41.3 ^a^	39.0 ^b^	0.770		
C18:1n9c (Oleic)				0.01	0.01
DIM 60	18.5 ^b^	24.7 ^a^	0.640		
DIM 75	16.9 ^b^	20.0 ^a^	0.640		
C21:0 (Henicosanoic)				0.01	0.02
DIM 60	0.78 ^a^	0.65 ^b^	0.026		
DIM 75	0.80 ^a^	0.64 ^b^	0.025		
∑ Saturated fatty acids (SFA)				0.05	0.01
DIM 60	77.0 ^a^	70.5 ^b^	0.895		
DIM 75	78.6	75.6	0.908		
∑ Unsaturated fatty acids (UFA)				0.02	0.01
DIM 60	22.4 ^b^	29.4 ^a^	0.908		
DIM75	21.3	24.2	0.903		
∑ Monounsaturated fatty acids (MUFA)				0.01	0.01
DIM 60	20.1 ^b^	26.4 ^a^	0.878		
DIM 75	18.6	21.7	0.872		
UFA/SFA				0.05	0.01
DIM 60	0.30 ^b^	0.42 ^a^	0.017		
DIM 75	0.27 ^b^	0.32 ^a^	0.016		
Mid-lactation					
C16:0 (Palmitic)				0.25	0.02
DIM 120	50.78	49.53	0.68		
DIM 135	51.69 ^a^	48.5 ^b^	0.65		
C18:1n9c (Oleic)				0.11	0.03
DIM 120	15.68	15.76	0.29		
DIM 135	14.68 ^b^	16.75 ^a^	0.27		

^a,b^ Differs (*p* ≤ 0.05) between treatments (lines). DIM: days in milk; ∑sum of fatty acids; ω: omega. ^1^ The phytogenic additive is a combination of cinnamon and oregano essential oil, chelated amino acid chromium, selenium proteinate, inactivated *Saccharomyces cerevisiae*, *S. cerevisiae*, turmeric extract, and tannic acid.

## Data Availability

Raw data are held by the authors and may be available upon request.

## Supplementary Materials

The following supporting information can be downloaded at: https://www.mdpi.com/article/10.3390/ani14172518/s1, S1: Illustration of the feeders and drinkers used during the cows’ holding period; S2: Standardization of methodology for evaluating fatty acids in the rumen of cows that consumed the feed additive; S3: Profile of fatty acids in the Total Mixed Ration (TMR) of dairy cows during early lactation (30–75 days in milk) and mid-lactation (90–135 days in milk); S4: Treatment × day interaction for milk production (mean and standard deviation) of dairy cows during early lactation (30–75 days in milk) and mid-lactation (90–135 days in milk). Note: Phytogenic group: animals’ intake an additive formulated with the combination of cinnamon and oregano essential oil, chelated amino acid chromium, selenium proteinate, inactivated *Saccharomyces cerevisiae*, *S. cerevisiae*, turmeric extract, and tannic acid. * *p* ≤ 0.05; S5: Treatment × day interaction for immunoglobulin A (IgA), ceruloplasmin (CERU) and haptoblobin (HATP) of dairy cows during early lactation (30–75 days in milk) and mid-lactation (90–135 days in milk): Control (CON) vs. Phytogenic (PHY); S6: Treatment × day interaction for oxidative status (SOD: superoxide dismutase; GST: Glutathione S-transferase; TBARS: lipid peroxidation; CAB-P: cabonyl protein) of dairy cows during early lactation (30–75 days in milk) and mid-lactation (90–135 days in milk): Control (CON) vs. Phytogenic (PHY); S7: Profile of fatty acids in milk of dairy cows during early lactation (30–75 days in milk) and mid-lactation (90–135 days in milk: Control (CON) vs. Phytogenic (PHY); S8: Beta diversity of microorganisms in the milk of dairy cows during early lactation (30–75 days in milk) and mid-lactation (90–135 days in milk: Control (CON) vs. Phytogenic (PHY). Note: Phytogenic group: animals’ intake an additive formulated with the combination of cinnamon and oregano essential oil, chelated amino acid chromium, selenium proteinate, inactivated *Saccharomyces cerevisiae*, *S. cerevisiae*, turmeric extract, and tannic acid.
