# Supplementary material for: Combination of Phytoactives in the Diet of Lactating Jersey Cows: Effects on Productive Efficiency, Milk Composition and Quality, Ruminal Environment, and Animal Health
Source: Animals (Basel). 2024 Aug 29;14(17):2518. doi: 10.3390/ani14172518 (PMC11394032; doi:10.3390/ani14172518)

Supplementary material 1 (S1). Illustration of the feeders and drinkers used during the cows' holding period.

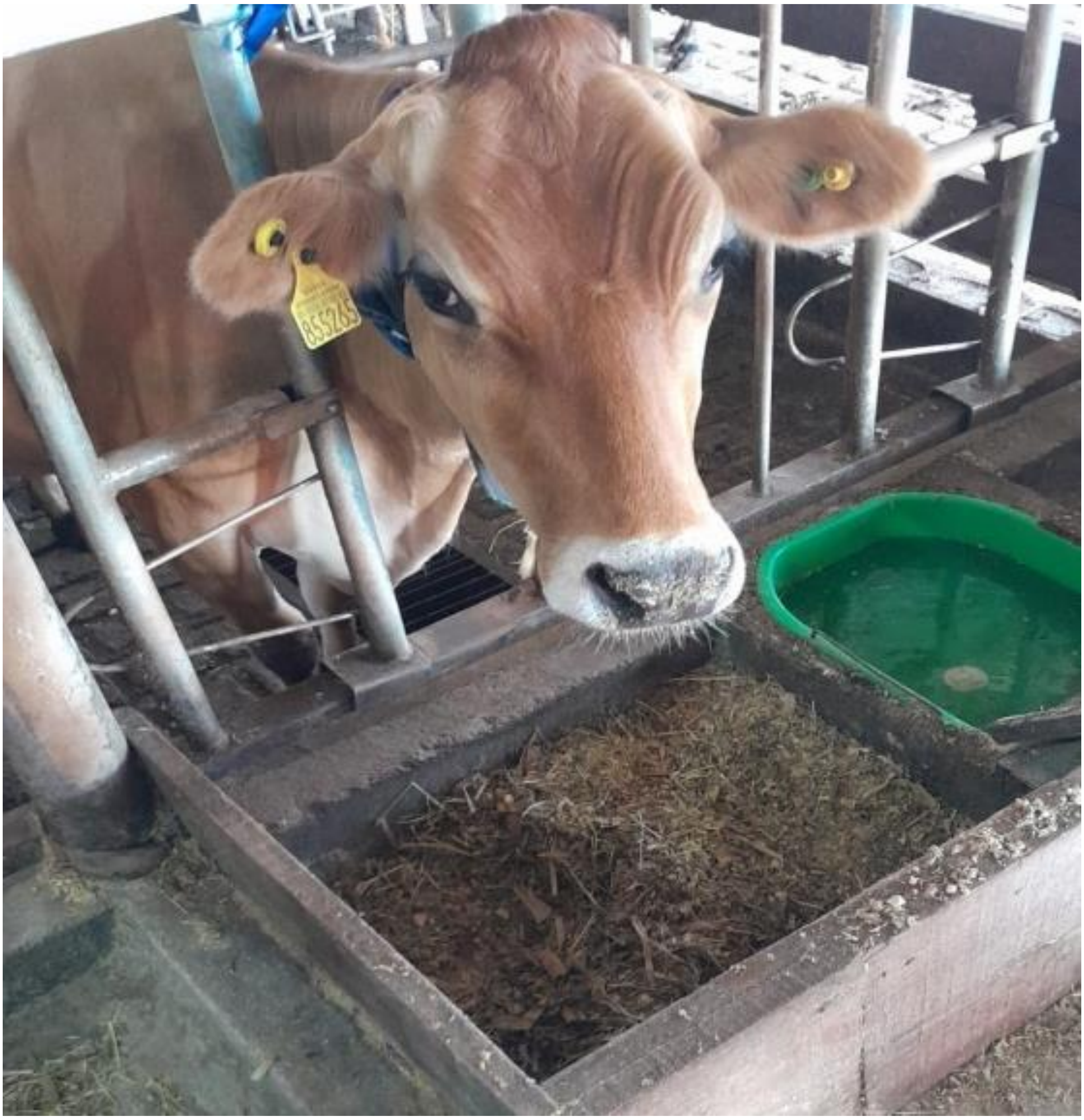

Supplementary Material 2 (S2). Standardization of methodology for evaluating fatty acids in the rumen of cows that consumed the feed additive.

	Acetic acid	Propionic acid	Butyric acid	Isovaleric acid
**R^2**	0.9995	0.9997	0.9996	0.9996
**Equation**	y = 0.0095x + 0.0061	y = 0.0158x + 0.0046	y = 0.0238x - 0.0049	y = 0.0304x - 0.0029
**Linear range (mmol L^{-1})***	2.22 - 133.01	1.69 - 101.24	1.40 - 56.01	0.57 - 13.57
**LOD (mmol L^{-1})**	1.11	0.84	0.70	0.28
**LOQ (mmol L^{-1})**	2.22	1.69	1.40	0.57
**Accuracy**	105.95	107.97	103.82	100.93
**Repeatability (RSD)**	2.16	2.16	1.86	1.77

* The linear range, LOD (limit of detection), and LOQ (limit of quantitation) were expressed in mmol of SFA for L of ruminal fluid

Supplementary material 3 (S3): Profile of fatty acids in the Total Mixed Ration (TMR) of dairy cows during early lactation (30-75 days in milk) and mid-lactation (90-135 days in milk)

**Compounds – fatty acid**	TMR: peak lactation		TMR: average lactation	
	Control	Phytogenic[1]	Control	Phytogenic[1]
C10:0 (Capric)	0.045	0.039	-	-
C12:0 (Lauric)	0.160	0.143	0.200	0.205
C14:0 (Myristic)	0.414	0.376	0.512	0.481
C15:0 (Pentadecanoic)	0.310	0.307	0.206	0.194
C16:0 (Palmitic)	19.789	20.178	28.4	26.1
C16:1 (Palmitoleic)	0.298	0.302	0.195	0.176
C17:0 (Heptadecanoic)	0.259	0.263	0.312	0.318
C18:0 (Stearic)	5.106	5.476	5.94	5.64
C18:1n9c (Oleic)	27.285	27.334	23.09	23.11
C18:2n6c (Linoleic)	38.237	37.735	33.1	35.6
C20:0 (Arachidic)	0.673	0.684	0.86	0.84
C20:1n9 (cis-11-Eicosenoic)	0.307	0.302	0.25	0.26
C18:3n3 (a-Linolenic)	5.355	5.069	3.92	4.46
C21:0 (Henicosanoic)	0.076	0.073	-	-
C20:2 (cis-11.14-Eicosadienoic)	0.120	0.162	-	-
C22:0 (Behenic)	0.770	0.750	0.95	0.88
C22:1n9 (Erucic)	0.028	0.036	0.10	0.09
C20:3n3 (cis-11.14.17-Eicosatrienoic)	0.009	0.009	-	-
C20:4n6 (Arachidonic)	0.164	0.157	0.26	0.26
C24:0 (Lignoceric)	0.595	0.605	0.94	0.92
Other variables				
∑ Saturated fatty acids (SFA)	28.197	28.895	38.1	35.5
∑ Unsaturated fatty acids (UFA)	71.803	71.105	61.8	64.2
∑ Monounsaturated fatty acids (MUFA)	27.918	27.973	24.2	24.15
∑ Polyunsaturated fatty acids (PUFA)	43.885	43.131	37.5	40.3
UFA/SFA	2.546	2.461	1.61	1.81
∑ ω6	38.401	37.892	33.5	38.5
∑ ω3	5.364	5.077	3.90	4.46
ω6/ω3	7.160	7.463	8.58	8.04

Note: Phytogenic group: animals' intake an additive formulated with the combination of cinnamon and oregano essential oil, chelated amino acid chromium, selenium proteinate, inactivated *Saccharomyces cerevisiae*, *S. cerevisiae*, turmeric extract, and tannic acid.

Supplementary material 4 (S4). Treatment x day interaction for milk production (mean and standard deviation) of dairy cows during early lactation (30-75 days in milk) and mid-lactation (90-135 days in milk). Note: Phytogenic group: animals' intake an additive formulated with the combination of cinnamon and oregano essential oil, chelated amino acid chromium, selenium proteinate, inactivated *Saccharomyces cerevisiae*, *S. cerevisiae*, turmeric extract, and tannic acid. * P ≤ 0.05.

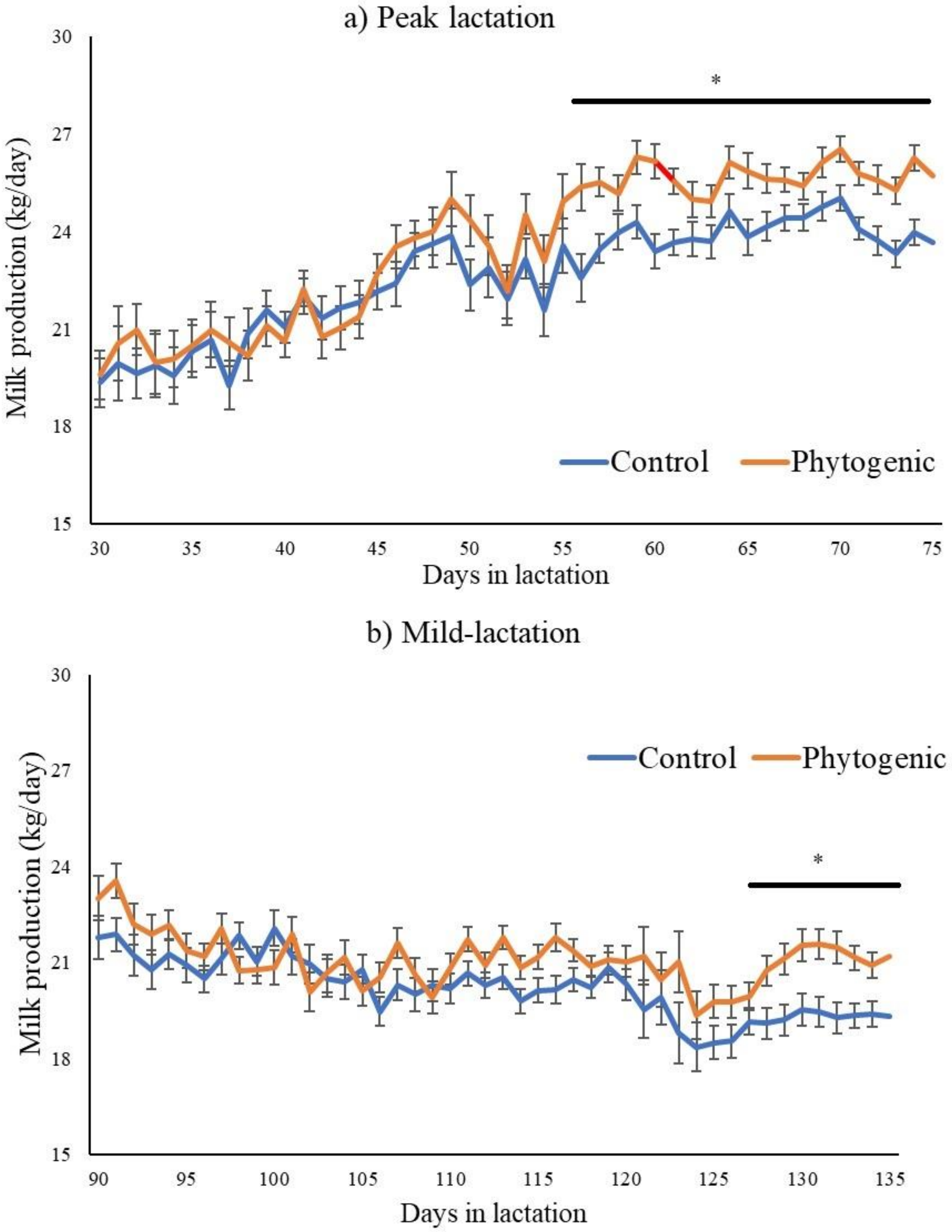

Supplementary material 5 (S5). Treatment x day interaction for immunoglobulin A (IgA), ceruloplasmin (CERU) and haptoblobin (HATP) of dairy cows during early lactation (30-75 days in milk) and mid-lactation (90-135 days in milk): Control (CON) vs. Phytogenic (PHY).

Exp. period	IgA	CERU	HAPT
Peak lactation	CON/PHYT	CON/PHYT	CON/PHYT
DIM 30	0.76 / 0.75	0.74 / 0.73	0.24 / 0.24
DIM 45	0.79 / 0.89*	0.78 / 0.51 *	0.26 / 0.23
DIM 60	0.77 / 0.84*	0.75 / 0.46 *	0.24 /0.21
DIM 75	0.78 / 0.80	0.76 / 0.38 *	0.25 / 0.14*
Average lactation			
DIM 90	-	0.85 / 0.47*	0.25 / 0.16*
DIM 105	-	0.84 / 0.45*	029 / 0.18*
DIM 120	-	0.81 / 0.53*	0.31 / 0.21*
DIM 135	-	0.77 / 0.58*	0.33 / 0.22*

Note: Asterisk (*) illustrates the difference in the interaction between treatment × day ($P \leq 0.02$). Note: Phytogenic group: animals' intake an additive formulated with the combination of cinnamon and oregano essential oil, chelated amino acid chromium, selenium proteinate, inactivated *Saccharomyces cerevisiae*, *S. cerevisiae*, turmeric extract, and tannic acid.

Supplementary material 6 (S6). Treatment x day interaction for oxidative status (SOD: superoxide dismutase; GST: Glutathione S-transferase; TBARS: lipid peroxidation; CAB-P: cabonyl protein) of dairy cows during early lactation (30-75 days in milk) and mid-lactation (90-135 days in milk): Control (CON) vs. Phytogenic (PHY).

Exp. period	SOD	GST	TBARS	CAB-P
Peak lactation	CON/PHY	CON/PHY	CON/PHY	CON/PHY
DIM 30	12.0 / 10.3	30.5 / 29.2	26.1 / 23.8	5.19 / 5.42
DIM 60	13.6 / 12.2	15.8 / 25.9*	34.8 / 16.9*	3.47 / 4.20
DIM 75	14.5 / 11.2*	17.8 / 20.9	41.7 / 29.6*	6.82 / 4.18*
Average lactation				
DIM 90	-	22.7 / 23.3	33.4 / 22.8*	-
DIM 120	-	13.0 / 22.8*	28.6 / 17.9*	-
DIM 135	-	17.9 / 22.7*	13.4 / 8.15*	-

Note: Asterisk (*) illustrates the difference in the interaction between treatment × day ($P \leq 0.03$). Note: Phytogenic group: animals' intake an additive formulated with the combination of cinnamon and oregano essential oil, chelated amino acid chromium, selenium proteinate, inactivated *Saccharomyces cerevisiae*, *S. cerevisiae*, turmeric extract, and tannic acid.

Supplementary Material 7 (S7): Profile of fatty acids in milk of dairy cows during early lactation (30-75 days in milk) and mid-lactation (90-135 days in milk: Control (CON) vs. Phytogenic (PHY).

**Fatty acid**	CON	PHY[2]	SEM	P treat[1]	P treat x day[1]
Early lactation					
C6:0 (Caproic)	0.69	0.64	0.025	0.74	0.59
C8:0 (Caprylic)	0.66	0.62	0.020	0.84	0.77
C10:0 (Capric)	2.21	2.08	0.076	0.66	0.71
C11:0 (Undecanoic)	0.17	0.16	0.008	0.88	0.92
C12:0 (Lauric)	3.10	2.96	0.115	0.41	0.25
C13:0 (Tridecanoic)	0.13	0.11	0.006	0.86	0.89
C14:0 (Myristic)	12.5	11.7	0.262	0.78	0.64
C14:1 (Myristoleic)	0.61	0.54	0.026	0.52	0.41
C15:0 (Pentadecanoic)	0.97	0.88	0.027	0.11	0.23
C16:1 (Palmitoleic)	0.87	0.92	0.037	0.79	0.62
C17:0 (Heptadecanoic)	0.57	0.60	0.013	0.92	0.87
C17:1 (cis-10-Heptadecenoic)	0.13	0.17	0.011	0.64	0.52
C18:0 (Stearic)	15.6	15.9	0.415	0.95	0.92
C18:2n6c (Linoleic)	2.37	2.41	0.073	0.93	0.89
C20:0 (Arachidic)	0.22	0.21	0.006	0.97	0.96
C18:3n6 (?-Linolenic)	0.02	0.02	0.001	0.99	0.99
C20:1n9 (cis-11-Eicosenoic)	0.06	0.06	0.003	0.98	0.97
C18:3n3 (a-Linolenic)	0.21	0.20	0.007	0.94	0.96
C20:2 (cis-11.14-Eicosadienoic)	0.02	0.02	0.001	0.98	0.99
C22:0 (Behenic)	0.11	0.10	0.003	0.98	0.97
C20:3n6 (cis-8.11.14-Eicosatrienoic)	0.08	0.07	0.003	0.95	0.97
C22:1n9 (Erucic)	0.01	0.01	0.003	0.99	0.99
C20:3n3 (cis-11.14.17-Eicosatrienoic)	0.00	0.01	0.002	0.98	0.93
C20:4n6 (Arachidonic)	0.03	0.04	0.008	0.97	0.92
C22:2 (cis-13.16-Docosadienoic)	0.01	0.01	0.003	0.99	0.98
C24:0 (Lignoceric)	0.05	0.06	0.013	0.94	0.96
C20:5n3 (cis-5.8.11.14.17-Eicosapentaenoic)	0.01	0.02	0.003	0.97	0.98
C24:1n9 (Nervonic)	0.01	0.01	0.003	0.99	0.99
∑ Polyunsaturated fatty acids (PUFA)	2.76	2.80	0.083	0.87	0.92
∑ ω6	2.51	2.54	0.076	0.75	0.86
∑ ω3	0.23	0.22	0.008	0.95	0.97
ω6/ω3	11.2	11.4	0.247	0.91	0.93
Mid-lactation					
C6:0 (Caproic)	0.63	0.70	0.02	0.79	0.55
C8:0 (Caprylic)	0.58	0.65	0.02	0.80	0.71
C10:0 (Capric)	2.02	2.33	0.08	0.46	0.38
C11:0 (Undecanoic)	0.20	0.24	0.01	0.87	0.80
C12:0 (Lauric)	3.04	3.62	0.08	0.18	0.21
C13:0 (Tridecanoic)	0.14	0.18	0.01	0.37	0.12

C14:0 (Myristic)	12.07	13.73	0.25	0.58	0.69
C14:1 (Myristoleic)	0.79	0.86	0.02	0.66	0.54
C15:0 (Pentadecanoic)	1.07	1.32	0.06	0.46	0.61
C16:1 (Palmitoleic)	1.29	1.31	0.04	0.92	0.90
C17:0 (Heptadecanoic)	0.51	0.58	0.01	0.20	0.11
C17:1 (cis-10-Heptadecenoic)	0.14	0.16	0.01	0.76	0.85
C18:0 (Stearic)	10.46	11.11	0.36	0.50	0.24
C18:2n6c (Linoleic)	1.92	1.97	0.03	0.94	0.89
C20:0 (Arachidic)	0.15	0.16	0.01	0.91	0.95
C18:3n6 (?-Linolenic)	0.02	0.02	0.00	0.98	0.96
C20:1n9 (cis-11-Eicosenoic)	0.04	0.04	0.00	0.97	0.96
C18:3n3 (a-Linolenic)	0.18	0.19	0.01	0.95	0.97
C21:0 (Henicosanoic)	0.26	0.28	0.01	0.91	0.83
C20:2 (cis-11.14-Eicosadienoic)	0.03	0.03	0.00	0.99	0.97
C22:0 (Behenic)	0.06	0.06	0.00	0.94	0.97
C20:3n6 (cis-8.11.14-Eicosatrienoic)	0.08	0.08	0.00	0.99	0.95
C20:4n6 (Arachidonic)	0.03	0.03	0.00	0.98	0.98
C24:0 (Lignoceric)	0.04	0.05	0.00	0.97	0.94
C20:5n3 (cis-5.8.11.14.17-Eicosapentaenoic)	0.01	0.01	0.00	0.99	0.99
C24:1n9 (Nervonic)	0.01	0.01	0.00	0.99	0.98
∑ Saturated fatty acids (SFA)	82.77	84.69	1.02	0.25	0.18
∑ Unsaturated fatty acids (UFA)	19.73	20.96	0.27	0.53	0.15
∑ Monounsaturated fatty acids (MUFA)	17.45	18.62	0.22	0.62	0.31
∑ Polyunsaturated fatty acids (PUFA)	2.28	2.33	0.06	0.38	0.12
UFA/SFA	0.25	0.26	0.05	0.97	0.90
∑ ω6	2.05	2.11	0.09	0.98	0.94
∑ ω3	0.19	0.20	0.02	0.96	0.98
ω6/ω3	10.68	11.25	0.19	0.42	0.20
∑ ω6	2.05	2.11	0.09	0.55	0.41

Note 1: There is no statistical difference between groups or interaction between treatments ($P \leq 0.05$), as well as any trend (>0.05 and ≤ 0.10) for these fatty acids in the milk of cows in the beginning and middle of lactation. Note 2: Phytogenic group: animals' intake an additive formulated with the combination of cinnamon and oregano essential oil, chelated amino acid chromium, selenium proteinate, inactivated *Saccharomyces cerevisiae*, *S. cerevisiae*, turmeric extract, and tannic acid.

Supplementary material 8. Beta diversity of microorganisms in the milk of dairy cows during early lactation (30-75 days in milk) and mid-lactation (90-135 days in milk: Control (CON) vs. Phytogenic (PHY). Note: Phytogenic group: animals' intake an additive formulated with the combination of cinnamon and oregano essential oil, chelated amino acid chromium, selenium proteinate, inactivated *Saccharomyces cerevisiae*, *S. cerevisiae*, turmeric extract, and tannic acid.

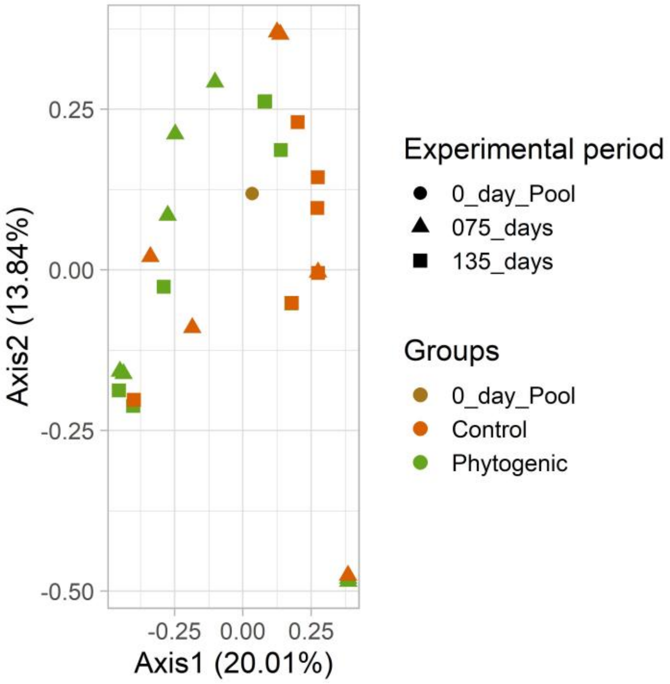

Supplement: Supplementary file 1 [file animals-14-02518-s001.zip › animals-3073942-supplementary.pdf]
